# Prenatal onset of the neuroradiologic phenotype of pyruvate carboxylase deficiency due to homozygous *PC* c.1828G > A mutations

**DOI:** 10.1002/jmd2.12235

**Published:** 2021-06-14

**Authors:** Aizeddin A. Mhanni, Cheryl Rockman‐Greenberg, Lawrence Ryner, Martin Bunge

**Affiliations:** ^1^ Department of Pediatrics and Child Health Max Rady College of Medicine, Rady Faculty of Health Sciences Winnipeg Canada; ^2^ Research Institute in Oncology and Hematology, Cancer Care Manitoba Winnipeg Canada; ^3^ Department of Radiology, Rady Faculty of Health Sciences, Max Rady College of Medicine University of Manitoba Winnipeg Canada

**Keywords:** brain morphogenesis, lactic acidosis, neuroimaging, prenatal onset, pyruvate carboxylase

## Abstract

Pyruvate carboxylase (PC) deficiency (MIM# 266150) is an autosomal recessive disorder with three subtypes. Patients homozygous for the c.1828G > A mutation in the *PC* gene belong to type A, which typically has infantile onset, severe to profound developmental delay, hypotonia, and lactic acidemia. We report the neuroimaging abnormalities in a 33‐week gestation infant homozygous for the c.1828G > A mutation. Brain magnetic resonance imaging on day 10 of life revealed increased T2 signal within the subcortical and periventricular white matter, an immature gyral pattern, large periventricular cysts with mass effect on the lateral ventricles, and dilatation of the occipital and temporal horns. Magnetic resonance spectroscopy showed reduced creatine and NAA peaks, a relatively high choline peak and no lactate peak. These findings were observed prior to the neonate experiencing any episodes of decompensation with lactic acidosis. The presence of these brain anomalies at this gestational age, prior to any metabolic decompensation, supports the essential role of PC in normal brain morphogenesis and the resulting in‐utero brain anomalies secondary to its deficiency. Our experience with this affected premature infant and many others we have managed with the same founder mutation suggests that the clinical phenotypes of the type A and the more severe type B PC deficient patients are on a spectrum rather than distinct subtypes.

**Synopsis**

In‐utero onset of brain anomalies in pyruvate carboxylase deficiency.

## 1 INTRODUCTION

Pyruvate carboxylase (PC) is a biotin‐containing enzyme that is responsible for the adenosine triphosphate (ATP)‐dependent carboxylation of pyruvate to oxaloacetate, a key intermediate in the tricarboxylic acid cycle (CAC).^1^, ^2^ In mammals, PC is located solely in the mitochondrial matrix.^3^ The roles of PC in intermediate metabolism are tissue specific. In gluconeogenic tissues (liver and kidney), it catalyzes the first step in the synthesis of glucose from pyruvate, whereas in lipogenic tissues (liver, adipose, lactating mammary gland, and adrenal gland), it participates in the export of acetyl groups, as citrate, from the mitochondria to the cytosol. In central nervous system (CNS) tissues, PC participates in the replenishment of neurotransmitter pools of glutamate, gamma‐aminobutyric acid (GABA), and aspartate.^4^

PC deficiency is a rare autosomal recessive disease, characterized by impairment of gluconeogenesis and lactate metabolism, producing severe lactic acidosis.^5^ The primary result of the defect is a major deficit of oxaloacetate for the CAC.^6^ This leads to profound energy deficiency due to compromised function of the CAC.

Based on the severity of the clinical presentation and the biochemical disturbances, three phenotypes have been identified. Type A or “North American phenotype” is associated with profound developmental delay, lactic acidemia, shortened life span but with a normal lactate to pyruvate ratio. Type B or “French phenotype” presents neonatally, or in early infancy, with severe metabolic ketoacidosis, lactic acidosis (elevated lactate to pyruvate ratio), hyperammonemia, hepatomegaly, and delayed myelination. These patients usually die within the first weeks of life. The third phenotype, type C, presents with metabolic acidosis, normal growth and either normal psychomotor development or mild delay.

Type A PC deficiency is overrepresented in the North American Indigenous population, specifically the Ojibwa and Cree populations in Northwestern Ontario and Northeastern Manitoba, Canada, with an estimated carrier frequency as high as 1 in 10.^5^, ^7^ One homozygous mutation in the PC gene, c.1828 G > A (p.A610T), is present in all these reported patients with PC deficiency.^5^ All reported patients with this variant had infantile onset symptoms and death in infancy or early childhood with profound developmental delay.^5^, ^8^

Neuroradiologic abnormalities as detected by magnetic resonance imaging (MRI) in type B PC deficiency have been previously described.^9^ Cystic periventricular leukomalacia at birth was common, but some patients also had periventricular T2 hyperintensity and rarely subcortical T2 hyperintensity.^9^ In contrast, few case reports of neuroimaging findings in type A PC deficiency patients have been published.^10^ Neuroradiologic findings have included subdural effusions, ischemia‐like brain lesions and periventricular hemorrhagic cysts. Progressive cerebral atrophy and delayed myelination have also been reported.^10^ Here, we describe the neuroradiologic abnormalities in a 33‐week gestation premature infant, homozygous for the *PC* c.1828G > A mutation, adding further evidence to the in‐utero onset of the brain anomalies in this disorder, prior to any postnatal metabolic decompensation.

## 2 CASE REPORT

### 2.1 Case report

The infant was born to a couple who were second cousins of Indigenous descent. She was born at 33 weeks gestational age by cesarean section. Her Apgar scores were 9 and 9 at 1 and 5 minutes, respectively. Her birth weight was 2570 g (75th %ile), length 47 cm (75th%ile), and head circumference of 33.5 cm (90th%ile). She was well at birth. This was a twin, dichorionic diamniotic, pregnancy. Her co‐twin was healthy with appropriate growth parameters.

An elevated citrulline on MS/MS newborn screening was reported on day 3 of life. The citrulline was elevated at 236 umol/L (normal < 35, critically elevated > 100). Her capillary lactate was normal at 2.0 (N: 0.5‐2.3 mmol/L), and capillary blood gas was normal. Her ammonia, AST, ALT, GGT, and INR were normal. Given the elevated citrulline and the ethnic background of the parents, a diagnosis of PC deficiency was considered. Targeted mutation analysis for the *PC* c.1828G > A mutation revealed that the infant is homozygous for this founder mutation. By 2 weeks of age she was noted to have a persistently mildly elevated capillary lactate (average 3.2 mmol/L) with a compensated metabolic acidosis. Brain MRI was done to screen for structural brain abnormalities (section 2.2). She manifested severe developmental delay, seizures, and recurrent episodes of lactic acidosis requiring admissions to the hospital. At 3 years of age, she was nonambulatory and exhibited choreoathetotic movements of her hands with marked central hypotonia, and poor head control. She was able to recognize her care providers and smiled but did not use any words for communication. She was dependent on gastrostomy tube for feeding. She died at 3 years of age after an acute illness.

After discovery of the neuroimaging abnormalities in the proband shortly after birth, although the twin sibling did not have biochemical abnormalities on the newborn screen, given the a priori risk of having PC deficiency of 25%, the known variable expressivity of the clinical and biochemical manifestations early on, and the expected delay in obtaining the results of targeted mutation analysis, the family consented to a brain MRI and magnetic resonance spectroscopy (MRS). Neither infant required sedation nor anesthesia and the procedure was done with standard proper bundling and it was well tolerated by both twins. Molecular testing for the *PC* c.1828G > A mutation in the twin sibling later excluded this diagnosis.

### 2.2 Neuroimaging

**FIGURE 1:**
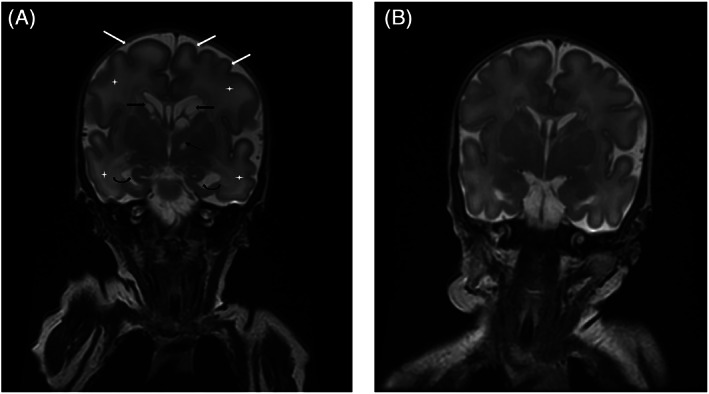
A, Coronal T2, TR:3450 TE:120.4. Day 10 of life, Pyruvate carboxylase (PC) deficient twin. Note the periventricular cysts (thick black arrows), cyst within the left globus pallidus (thin black arrow), enlarged temporal horns of the lateral ventricles (curved arrows), increased signal intensity of the white matter (white stars), shallow sulci and a simplified gyral pattern (white arrows). B, Coronal T2, TR:3450 TE:119.9. Unaffected twin sibling. Note the absence of periventricular cysts, normal white matter T2 signal, normal size of the lateral ventricles, and a normal gyration pattern for a neonate of gestational age 33 weeks

**FIGURE 2:**
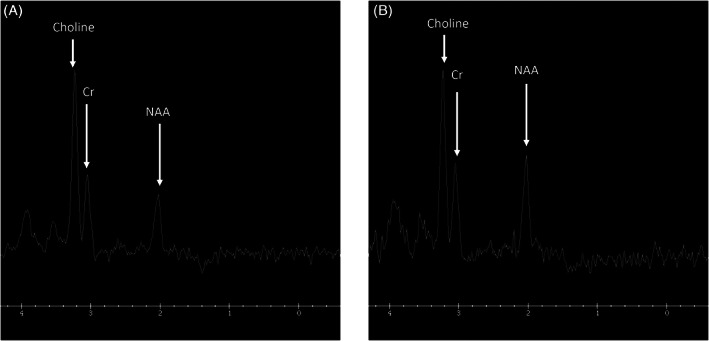
A, Magnetic resonance spectroscopy (MRS) (TR;1500/TE:144) with the voxel placed over the left basal ganglia. Day 10 of life in the pyruvate carboxylase (PC) deficient twin shows decreased creatine and N‐acetylaspartate (NAA). Peaks, no lactate peak. B, MRS (TR;1500/TE:144) with the voxel placed over the left basal ganglia. In the unaffected twin sibling shows a normal MRS spectrum for a neonate of gestational age 33 weeks

An MRI/MRS was obtained at 10 days of age (Figures 1A and 2A). This included sagittal T1 fluid‐attenuated inversion recovery, axial and coronal T2, axial T1, axial gradient echo, axial and coronal diffusion diffusion‐weighted imaging and trace images, single VOXEL MRS (TR;1500/TE:144) with the voxel placed over the left basal ganglia. The anatomic brain imaging with spectroscopy (MRS) was performed on a Siemens Magnetom 3 T Verio ‐ MR scanner. The twin sibling had the MRI/MRS examination at the same time on the same machine (Figures 1B and 2B).

The periventricular white matter and the subcortical white matter of the bilateral cerebral hemispheres appeared more hyperintense on the T2‐weighted sequences when compared to the imaging obtained on the twin sibling. This increased T2 hyperintensity relates to an increased water content within the white matter. This is thought to represent white matter edema. Alternatively, this may reflect prematurity of the white matter, since the gyral pattern of the affected twin showed shallow sulci and slightly less gyri than when compared to the unaffected twin.

The affected twin's imaging also showed multiple large periventricular cysts adjacent to the frontal horns and body of the lateral ventricles which have mass effect on the lateral ventricles. A smaller cyst without mass effect was present within the left globus pallidus.

Dilatation of the occipital horns and to a lesser extent temporal horns of the lateral ventricles was present in the affected twin, likely as a result of ex‐vacuo dilatation.

The MRS spectrum over the left basal ganglia demonstrates reduced creatine and N‐acetylaspartate (NAA) peaks. The choline peak was relatively high. There was no lactate peak (Figure 2A). Although the MRS technique used does not allow for absolute quantitation of the different metabolites of interest, relative ratios are calculated (Table 1). MR spectra were analyzed by taking the ratio of MRS peak heights (Myo‐Inositol (mI) at 3.55 ppm, choline (Cho) at 3.2 ppm, creatine (Cr) at 3.0 ppm, N‐acetyl aspartate (NAA) at 2.0 ppm, and lactate (Lac) at 1.33 ppm) and expressing the percent difference of the proband relative to the twin sibling. The MRS spectrum over the left basal ganglia demonstrates a reduced ratio of NAA to Cr peaks (NAA/Cr = 0.78 vs 1.13), compared to the unaffected twin, corresponding to a 31% reduction. The ratios of Cho/Cr and Cho/NAA were both elevated (Cho/Cr 2.38 vs 2.06, Cho/NAA 3.05 vs 1.83), compared to the unaffected twin, corresponding to a 16% and 67% increase, respectively. Lactate was not at sufficient concentrations to be detectable in the spectra. Correlation between the blood lactate levels and the brain lactate levels is not necessarily expected due to the low fractional blood volume within the MRS voxel (on the order of 5%).

**TABLE 1:** MRS metabolite relative levels and ratios in the twin siblings

MRS ratio	Affected twin	Unaffected twin	%difference
Col/Cr	2.38	2.06	+16
NAA/Cr	0.78	1.13	−31
Chol/NAA	3.05	1.83	+67
mI/NAA	0.41	0.43	−5
Lactate	ND	ND	

Abbreviation: MRS, magnetic resonance spectroscopy; ND, not detectable.

## 3 DISCUSSION

PC is a biotin‐containing mitochondrial enzyme that catalyzes the conversion of pyruvate to oxaloacetate. It plays an important role in gluconeogenesis and in energy production through replenishment of the Krebs cycle with oxaloacetate and in anaplerotic pathways such as neurotransmitter synthesis and lipogenesis. It is hypothesized that PC deficiency induces energy deprivation, reduces astrocytes' ability to protect against excitotoxic insults, and compromises microvascular autoregulation, leading to white matter injury.^10^ The presence of bilateral cysts adjacent to the lateral ventricles seen in PC deficiency is similar to descriptions of ischemic‐like brain lesions and periventricular leukomalacia in type B PC deficient patients.^9^ Cystic periventricular leukomalacia can also be seen in other inborn errors of metabolism such as oxidative phosphorylation disorders, pyruvate dehydrogenase (PDH) deficiency, isolated sulfite oxidase deficiency, molybdenum cofactor deficiency, and urea cycle disorders. Each of these disorders is associated with other distinctive clinical features allowing for a specific diagnosis.^11^

The presence of significant neuroimaging abnormalities in our patient at 33‐weeks gestation supports previous reports that the neurodevelopmental sequelae seen in this form of congenital lactic acidosis are of prenatal onset. Egloff et al. reported prenatal sonographic description of two affected fetuses of non‐Indigenous descent born to the same couple. A 32‐week gestation ultrasound showed bilateral paraventricular cysts, 19 mm on the right and 11 mm on the left. In the couple's subsequent pregnancy an ultrasound at 21‐week gestation was normal. At 25 week‐gestation, a follow‐up ultrasound showed three paraventricular cysts, two on the left, and one on the right side. An ultrasound at 33‐weeks gestation revealed bilateral subependymal cysts on the frontal horns, two on the right (7 and 10 mm) and one on the left (5 mm). Both infants were born at term and manifested severe neurologic dysfunction with severe lactic acidosis. One died at 6 days of age and the other at 40 days. A diagnosis of PC deficiency was confirmed in the two siblings molecularly.^12^ Although the exact mutation was not reported, the severe course of the disease and the non‐Indigenous ethnicity is supportive of type B PC deficiency. Brun et al. reported siblings with type B PC deficiency, one of whom was found to have increased head circumference and periventricular leukomalacia detected on fetal ultrasonography at 29.4‐weeks gestation.^13^ The brain anomalies previously described by Egloff et al and Brun et al further support that the neuroimaging abnormalities seen in our affected infant likely had their origin in the second trimester of pregnancy. Brain development becomes dependent on the enzymatic activity of PDH and PC activity during this period. Mortori et al recently showed that downregulation of PC expression exacerbates oxidative stress and accelerates neurodegeneration, pointing to the essential role of anaplerosis in the prevention of neurodegeneration.^14^ PC deficiency aggravates oxidative stress and neurodegeneration, compromising normal brain morphogenesis and resulting in neuronal damage.

The cystic leukomalacia noted in our patient is indicative of an early in utero insult prior to 32 weeks gestational age. Impaired synthesis of glutamine induced by PC deficiency deprives neurons of this critical amino acid leading to neuronal death secondary to decreased neurotransmitter pools of glutamate and GABA (^4^, ^15^, ^16^). The low NAA peak suggests neuronal loss and the high choline peak is indicative of white matter damage. The low creatine may reflect disrupted brain energy metabolism in PC deficiency. The MRS findings we present, with reduced creatine and NAA peaks, further support the hypothesis of an early prenatal brain insult with neuronal injury. The presence of these abnormalities prior to metabolic decompensations supports the hypothesis that the neurodevelopmental abnormalities seen in our patient only in part is due to the consequent recurrent metabolic decompensation characteristic of this disorder.

The neuroradiologic abnormalities we report at this gestational age, prior to any metabolic decompensation, thus support the essential role of the PC enzyme in normal brain morphogenesis during the first and second trimesters and the resulting in‐utero brain anomalies secondary to its deficiency. While type A PC deficiency patients who are homozygous for the c.1828G > A mutation have longer survival than type B patients, our experience with this affected premature infant and many others, we have managed with the same founder mutation suggests that the clinical phenotypes of type A and type B PC deficient patients are on a spectrum rather than distinct subtypes.

## CONFLICT OF INTEREST

The authors have no potential conflicts of interest to disclose.

## AUTHORS CONTRIBUTIONS

Aizeddin A. Mhanni, Cheryl Rockman‐Greenberg, and Martin Bunge shared conceptualization, methodology, data collection, and data analysis. Aizeddin A. Mhanni wrote the initial draft of the manuscript, and all authors participated in discussing and editing the manuscript. Lawrence Ryner and Martin Bunge analyzed and interpreted the MRS data.

## ETHICS STATEMENT

Formal ethics approval for publication was obtained from the Research Ethics Board of the University of Manitoba.

## A PATIENT CONSENT STATEMENT

The family has verbally consented to the use of de‐identified clinical information to be used for this case report.
